# European Laryngological Society position paper on laryngeal dysplasia Part II: diagnosis, treatment, and follow-up

**DOI:** 10.1007/s00405-020-06406-9

**Published:** 2020-10-14

**Authors:** Hans Edmund Eckel, Ricard Simo, Miquel Quer, Edward Odell, Vinidh Paleri, Jens Peter Klussmann, Marc Remacle, Elisabeth Sjögren, Cesare Piazza

**Affiliations:** 1 Department of Oto-Rhino-Laryngology, Klagenfurt General Hospital, Klagenfurt am Wörthersee, Austria; 2 Department of Otorhinolaryngology Head and Neck Surgery, Guy’s and St Thomas’ Hospital, London, UK; 3 Department of Otorhinolaryngology and Head and Neck Surgery, Hospital de la Santa Creu i Sant Pau. Universitat Autònoma de Barcelona, Barcelona, Spain; 4 Department of Head and Neck Pathology, King’s College London Guy’s Hospital, London, UK; 5 Department of Otorhinolaryngology Head and Neck Surgery, Royal Marsden Hospital, London, UK; 6 Department of Otorhinolaryngology, Head and Neck Surgery, Medical Faculty, University of Cologne, Cologne, Germany; 7 Department of Otorhinolaryngology, Head and Neck Surgery, CH Luxembourg, Luxembourg, Belgium; 8 Department of Otorhinolaryngology, Head and Neck Surgery, Leiden University Medical Center, Leiden, The Netherlands; 9 Department of Otorhinolaryngology-Head and Neck Surgery, ASST Spedali Civili of Brescia, University of Brescia, Brescia, Italy

**Keywords:** Carcinoma in situ, Dysplasia, Laryngeal intraepithelial neoplasia, Laryngeal carcinoma, Laser surgery, Radiotherapy

## Abstract

**Purpose of review:**

To give an overview of the current knowledge regarding the diagnosis, treatment, and follow-up of laryngeal dysplasia (LD) and to highlight the contributions of recent literature.

**Summary:**

The diagnosis of LD largely relies on endoscopic procedures and on histopathology. Diagnostic efficiency of endoscopy may be improved using videolaryngostroboscopy (VLS) and bioendoscopic tools such as Narrow Band Imaging (NBI) or Storz Professional Image Enhancement System (SPIES). Current histological classifications are not powerful enough to clearly predict the risk to carcinoma evolution and technical issues such as sampling error, variation in epithelial thickness and inflammation hamper pathological examination. Almost all dysplasia grading systems are effective in different ways. The 2017 World Health Organization (WHO) system should prove to be an improvement as it is slightly more reproducible and easier for the non-specialist pathologist to apply. To optimize treatment decisions, surgeons should know how their pathologist grades samples and preferably audit their transformation rates locally. Whether carcinoma in situ should be used as part of such classification remains contentious and pathologists should agree with their clinicians whether they find this additional grade useful in treatment decisions. Recently, different studies have defined the possible utility of different biomarkers in risk classification. The main treatment modality for LD is represented by transoral laser microsurgery. Radiotherapy may be indicated in specific circumstances such as multiple recurrence or wide-field lesions. Medical treatment currently does not have a significant role in the management of LD. Follow-up for patients treated with LD is a fundamental part of their care and investigations may be supported by the same techniques used during diagnosis (VLS and NBI/SPIES).

## Diagnostic procedures

The diagnosis of laryngeal dysplasia (LD) largely relies on endoscopic procedures and on histopathology. Imaging modalities (CT or MR scanning) may be obtained in cases with clinically unclear presentation, but have no role in the routine diagnostic process of suspected LD.

## Endoscopy

Various attempts have been made to verify whether videolaryngostroboscopy (VLS) may be accurate enough in distinguishing intraepithelial lesions from microinvasive or frankly invasive vocal fold lesions. At least from the theoretical point of view, this should be possible due to the well-known persistence of an undisturbed mucosal wave in presence of mild to severe dysplasia, with impairment of this physiological phenomenon when the neoplastic proliferation involves the vocal ligament, thus tethering the epithelium to the superficial layer of the lamina propria during phonation. However, a number of studies have found a suboptimal accuracy of VLS in performance of such a task, either due to concurrent inflammatory processes that may alter the mucosal wave even in the presence of intraepithelial diseases, or due to subjective variation of the subtle exam interpretation, suboptimal glottic plane visualization or other technical limits [1–3]. Attempts to correlate various vocal fold vibratory patterns with the nature of dysplasia (mild vs. severe) have also been frustrated [2]. However, VLS, especially in combination with videorecording and various bioendoscopic tools, may well improve diagnostic accuracy in LDs and their differential diagnosis from more aggressive lesions in respect to simple videolaryngoscopic evaluation under white light (WL).

As a matter of fact, a great help in trying to distinguish high- from low-risk lesions (i.e., lesions with a low malignant potential like keratosis without atypia or mild dysplasia) may be derived from the systematic in-office use of bioendoscopic techniques such as the Narrow Band Imaging (NBI) by Olympus or the Storz Professional Image Enhancement System (SPIES), that have been repeatedly demonstrated to be superior to WL endoscopy alone in giving a valuable insight into the biologic behaviour of LDs [4]. As recently demonstrated by Stanikova and coworkers, both methods yield comparable outcomes in distinguishing benign/low-risk from malignant/high-risk lesions, with a sensitivity and specificity of 83% and 98%, respectively, for NBI, and 86% and 96% for SPIES [5]. Both techniques rely on a widely-applied classification of neoangiogenetic vascular changes that, when present, have the aspect, defined by Arens and coworkers, of “perpendicular vessels”, indicative of a potentially malignant disease (from mild dysplasia upward) [6]. By contrast, when no vessels are visible or present a normal course (defined by Arens as “longitudinal”, i.e., parallel to the surface of the epithelial layer), this is strongly suggestive for a benign condition or a keratotic plaque without any malignant potential [6]. Clearly, these bioendoscopic signs may now help in choosing between a watchful waiting with regular follow-up versus immediate biopsy and/or resection, usually under general anaesthesia.

## Histopathological examination

Even leaving aside the fact that predicting the future is impossible, a number of practical problems make assessment of dysplasia and carcinoma risk in the larynx difficult. There is always a risk of sampling error, because the most marked histological changes do not necessarily occur in the red or thickly keratinised areas. Sampling to adequate depth is a particular problem when keratin is thick and the basal cell layers of the epithelium may be 2 mm or more below the surface. There are confusing variations in the normal microscopic anatomy of the cord. The epithelial thickness is very variable, from as few as 5 to over 20 cells in thickness in different places. This causes problems when dysplasia is graded in terms of the thickness involved. There is a complex transition between respiratory and squamous epithelium and occasionally glands within the cords, whose ducts can undergo metaplastic changes. Inflammation from reflux, possible candidal infection, and irradiation from previous cancer treatment further complicate the histological picture.

The published literature shows that almost all dysplasia grading systems differ but are effective in different ways [7]. The 2017 World Health Organization (WHO) system [8] should is slightly more reproducible and easier for the non-specialist pathologist to apply, but may be less specific that previous systems. Perhaps the most important point is that the surgeon should know how their pathologist grades samples and audit their transformation rate locally to calculate useful predictive values. It must be recognised that no grading systems are well designed and none account for the underlying biological process [9].

Although the 2017 WHO system has only been available for a short time, several studies have already challenged its definitions and reliability [4, 10] and it should be seen as an incremental improvement in a long process and not a perfect system.

Taken at a simple level, the WHO system seems straightforward to apply. The first decision is to determine whether invasion is present, itself often difficult in small fragmented and poorly orientated samples. Then it is necessary to decide whether mild changes can be accounted for by reactive benign processes rather than mild dysplasia, which can be difficult as no distinguishing criteria are provided. If these decisions are not possible, the sample may be unsuitable for grading. Grades may then be applied on the basis of the thickness of the epithelium involved, proliferation and maturation. Keratin is excluded, but whether terminally differentiated epithelium should also be included in differentiated lesions is unclear. Provided the other criteria given in Table 1 are met, a grade should be relatively easy to apply, given that there are only two. Unfortunately, what to do when the additional criteria listed are not met is unclear and causes most of the problems. All studies of grading systems have shown the importance of inter-examiner standardisation and training. Applying the system with a second experienced pathologist is essential to application of the system, to determine how such inconsistencies can be resolved and photographic standards would be far more helpful than text descriptions. Unfortunately, the table published in the WHO bluebook in 2017 contains an error that will cause some confusion in implementing the new system [4]. The amended correct form is shown in Table 1.

**Table 1:** Comparison of grading systems for LD, after WHO 2017 [8], with corrected levels. The published version limits low-grade dysplasia to the lower third, rather than the lower half of the epithelium

Level of abnormal maturation (WHO 2005)	WHO 2005 classification [61]	SIN classification [60]	Ljubljana classification [59]	Amended Ljubljana classification [58]	WHO 2017 [8]
	Squamous hyperplasia	Squamous hyperplasia	Squamous hyperplasia	Low-grade SIL	Low-grade dysplasia
Lower 1/3	Mild dysplasia	SIN 1	Basal/parabasal hyperplasia		
1/3 to 1/2	Moderate Dysplasia	SIN 1 or 2	Atypical hyperplasia	High-grade SIL	
Upper 1/2–3/4		SIN 2			High-grade dysplasia*
Full thickness	Severe dysplasia				
	Carcinoma in situ		Carcinoma in situ	Carcinoma in situ	

^*^A grade of carcinoma in situ may be used if a three-tiered system is preferred

*SIN*  squamous intraepithelial neoplasia, *SIL*  intraepithelial lesion

Whether carcinoma in situ should be used remains contentious and pathologists should agree with their clinicians whether they find this additional grade useful in treatment decisions. In our service, the grade is not normally used, but may be applied after discussion by the multidisciplinary team as it may trigger oncological treatment. Many published studies suggest that the additional grade provides useful predictive information [7].

## Biomarkers

Due to the limited prognostic value of the histological factors, biomarkers have long been investigated to try and better assess the risk of LD progressing to cancer. A systematic review published in 2012 showed that, until that moment, no marker or group of markers had been able to reliably indicate the risk of progression to carcinoma. Since then some new data has been published which show more promising results [11].

In 2014, a study was published concluding that chromosome instability was associated with malignant progression in LD [12]. In this study, neither histopathology nor the protein markers predicted progression in univariate analysis, although histopathological diagnosis, p53, and the apoptotic adaptor protein FADD contributed positively to chromosome instability in multivariate analysis. Chromosome instability was associated with malignant progression especially in lower grade lesions.

More recently, NANOG (a master regulator of embryonic stem cell pluripotency found to be frequently aberrantly expressed in a variety of cancers, including laryngeal SCC) has been proposed as a predictor of cancer progression for LD [13]. In this study, NANOG protein expression was evaluated by immunohistochemistry using two independent cohorts of patients with LD and correlated with clinicopathological parameters and laryngeal cancer risk. NANOG expression was detected by immunohistochemistry in 49 (60%) of 82 LD, whereas expression was negligible in patient-matched normal epithelia. Strong NANOG expression was found in 22 (27%) lesions and was established as cut-off point, showing the most robust association with laryngeal cancer risk (*p* = 0.003) superior to the histological classification (*p* = 0.320). Similar trends were obtained using a multicenter validation cohort of 86 patients with LD. These findings suggest that the use of NANOG expression may be of interest as a biomarker for cancer risk assessment in LD.

In a very recent publication, mutational profiling was studied in LD. Using targeted next-generation sequencing, non-synonymous mutations in six genes (PIK3CA, FGFR3, TP53, JAK3, MET, and FBXW7) were identified and validated by Sanger sequencing and/or qPCR. Mutations in PIK3CA and FGFR3 were detected in LD progressing to cancer but were absent in non-progressing cases. In contrast, mutations in JAK3, MET, and FBXW7 were found in non-progressing LDs but not in progressing cases. Except for R248W, mutations were mutually exclusive [14]. Moreover, five of seven progression dysplasia mutations were located in motif H2 of p53, whereas none of the non-progressing dysplasia mutations were. In summary, the authors proposed that the mutational profile of LD can be useful for the early detection of patients at risk of progression.

The studies above reinforce the concept that LD is a complex and multifaceted entity that encompasses different lesions with varying ability to evolve into carcinoma. Current histological classifications are not powerful enough to clearly predict the risk to carcinoma evolution. Recently, different studies have defined the possible utility of different biomarkers: chromosome instability, NANOG, and mutational profiles have been able to predict this risk more accurately and have demonstrated their practical utility.

## Natural course, recurrence rates, progression to infiltrating carcinoma

Isenberg et al. evaluated 2188 biopsies from laryngeal leukoplakia and identified mild or moderate dysplasia in 33.5% and severe dysplasia or carcinoma in situ in 15.2% [15]. Only histological evaluation could settle a final diagnosis as to the presence or absence of dysplasia. Laryngoscopy alone or in combination with VLS could not reliably allow for a final diagnosis. During follow-up (1–233 months) infiltrating laryngeal SCC was diagnosed in 3.7% of patients with no dysplastic lesions initially, 10.1% of patients with mild or moderate dysplasia, and 18.1% of patients with severe dysplasia. Karatayli-Ozgursoy et al. described a 15.9% transformation rate to invasive carcinoma for mild dysplasia in a 20-year single institution review [16]. A 12.1% rate for moderate and a 23.4% rate for severe dysplasia were noted. Transformation occurred in 48.6% of carcinoma in situ.

In contrast, the risk for developing laryngeal SCC out of LD showed no statistical correlation to the initial dysplasia grade in a recent review on 70 patients [17]. On average, malignant conversion took 127 weeks, 117 for mild dysplasia, 135 weeks for moderate dysplasia, and 82 weeks for severe dysplasia. The authors concluded that patients with LD are an inhomogeneous group and, as already stated above, the grade of LD alone seems to be an insufficient prognostic factor for the development of laryngeal cancer.

It is presumed that LD have a higher rate of malignant transformation compared to normal epithelium. The underlying concept is that consecutive mutations in normal epithelial cells lead to nuclear abnormalities, such as mitoses in the higher layers of the epithelium, loss of maturation, and architectural changes. These cellular abnormalities are termed dysplasia. Dysplasia can lead to malignant transformation over time [18]. Van Hulst et al. specifically studied the correlation between the grade of dysplasia and development of invasive laryngeal cancer in a systematic review of the medical literature [7]. They concluded rates of malignant transformation in mild dysplasia range from 0 to 41.7%, in moderate dysplasia from 0 to 48.0%, in severe dysplasia from 14.3 to 44.4%, and in carcinoma in situ from 11.1 to 75%. The authors found a wide variety in progression intervals between publications. Importantly, they found moderate dysplasia was more susceptible to malignant transformation than previously thought.

## Treatment

The variety of interventions reported and the lack of clarity regarding interventions and follow-up regimes have made the management of dysplasia very controversial [19]. Most of our current knowledge on the treatment of LD has been derived from publications with a focus on early stage glottic carcinomas. These results are frequently applied to LD patients, presuming that early invasive cancer and LD will react similarly to different treatments. Although this assumption may not be entirely justified, most of our understanding of LD treatment will have to rely on data obtained from early stage infiltrating cancer, until more relevant and specific data for LD become available.

The great majority of patients treated with LD have been treated with surgical methods. However, a minority will be treated with radiotherapy, for different reasons (usually, repeatedly recurring dysplasia) [16, 19, 20]. In most European institutions, transoral laser microsurgery is now considered the standard of care.

## Transoral laser microsurgery

While different types of lasers have been used in laryngeal surgery, the CO_2_ is still the undisputed workhorse for microlaryngeal surgery. Owing to its frequent use in tumour surgery, it is available in the Otolaryngology—Head and Neck Surgery Departments of most hospitals. The CO_2_ laser meets the requirements for use for most benign and malignant laryngeal lesions. In the American literature, angiolytic lasers have repeatedly been recommended for laryngeal surgery [21]. However, they have not found wider acceptance in Europe so far and large comparison studies demonstrating their superiority are still lacking. Additional equipment is available for the optimal delivery of laser energy to the operative site, including devices for focusing the surgical beam to an extremely small spot for precision cutting. Scanner systems are also available for coagulating predefined mucosal areas while preserving the underlying tissue. Transoral laser microsurgery (TLM) has become the most widely used therapeutic approach for laryngeal keratosis, dysplasia, carcinoma in situ, and early stage laryngeal squamous cell carcinoma [22, 23]. The results have been documented in numerous retrospective and some prospective case series [24]. It is generally used as a single modality treatment with curative intent and results in terms of local control rates are comparable to both open surgery and radiation therapy, with significantly less expenses. Immediate as well as long-term morbidities were also reduced when compared to both of them.

Laryngeal exposure is one of the most important factors influencing TLM resection of glottic cancer within safe surgical margins [25]. Anterior commissure involvement is associated with an increased risk of recurrence and possible deterioration of the voice quality [26–28]. Type 1 or 2 resections (according to the European Laryngological Society [ELS] classification of different transoral cordectomies [29]) are usually performed.

In their literature review on the management of LD, Sadri et al. found a pooled overall local control rate of 81% for TLM, as opposed to 91% for RT in treatment of severe dysplasia and carcinoma in situ of the larynx [19]. In contrast, Peretti et al. in their retrospective single institution review of TLM by CO_2_ laser in 71 patients reported a 5-year disease-specific survival, local control with laser alone, loco-regional, regional control, and organ preservation rates of 100%, 93.4%, 100%, 100%, and 98.5%, respectively [30].

From these data, it is obvious that the results of CO_2_ laser surgery have improved over time. It is also obvious that the results of any given surgical procedure will largely depend on the experience of the individual surgeon and the disease-specific workload of the institution delivering specialised services in the head and neck oncology and laryngology fields.

While RT gave higher local control rates than laser surgery when used as the initial treatment in the past, TLM can be used repeatedly in case of local recurrence, and the final laryngectomy-free survival rates seem to be higher with initial laser surgery as compared to radiation therapy (obviously a consequence of better re-treatment options in case of new dysplastic manifestations after primary treatment) [31, 32]. Although these findings are based primarily on the results for early infiltrating carcinoma, it is reasonable to believe that the same outcome can be expected for high-grade dysplasia and carcinoma in situ.

The need for a systematic second look microlaryngoscopy (SLM) under general anaesthesia at 6–12 weeks after the first procedure remains highly controversial. It can be dictated by uncertain (close or altered for iatrogenic artefacts) surgical margins, granulomas mimicking persistent/recurrent lesions, web formation or other post-excisional abnormal tissue growth at the level of the primary resection site potentially impacting on functional outcomes in spite of appropriate medical treatment and voice therapy, or involvement of certain laryngeal subsites (anterior commissure, ventricle, subglottis) [33]. The timing of SLM is still matter of discussion, being variably reported in the literature as ranging from 1 to 8 months after primary surgery. The same is true for the need of a third- or further-look microlaryngoscopy. However, both prospective and retrospective series clearly demonstrate a benefit in terms of earlier detection of persistence/recurrence (even in the presence of previous microscopically free surgical margins). This is especially true in case of anterior commissure involvement, and apparently normal larynx at fiberoptic evaluation [33, 34]. Moreover, an adjunctive advantage of SLM is the possibility to confirm the benign nature of symptomatic granulomas or webs and to promote their prompt surgical management with a functional gain in terms of voice and/or airway patency.

## Radiotherapy

Radiotherapy (RT) has also been employed in the management of LD. It has been used mainly on patients with high grade or severe dysplasia but its use and indications have remained controversial. It should be considered in patients who have multiple sites or field change in which surgical excision could lead to significant scarring and sequelae [35]. The main indications for RT in LD are summarised in Table 2.

**Table 2:** Indications for the management of LD with radiotherapy

1	Multiple recurrences
2	Persistent smoking and inability to cease habit
3	High anaesthetic risk
4	Patient’s preference
5	Wide-field multiple lesions

RT regimes used are similar to those applied for T1a carcinomas of the larynx [19]. The risks of progression to invasive carcinoma in patients treated by RT for severe dysplasia or carcinoma in situ has been reported to be around 12% [19]. Local control rates (LCR) range from 59 to 100%. Most recent series offer LCR between 79 and 98% [36, 37]. Patients most at risk are those who continue to smoke and drink and those who have lesions in the anterior commissure [38].

The main rationale for many authors for the use of RT has been the voice outcomes after such treatment. Persistent dysphonia has been found more frequently following TLM than after RT. Also on stroboscopic examination, incomplete glottic closure and diminution or lack of vibration of the operated vocal fold have been frequently observed following TLM. However, when maximum phonation time, mean airflow rate, fundamental frequency range of phonation, and intensity range of phonation have been analysed side to side no differences have been found [39]. RT is now considered a treatment option for severe or high-grade LD in a limited subset of patients with repeated recurrences, wide-field lesions, and high anaesthetic risks.

## Conventional microlaryngoscopic surgery

Apart from laser surgery, resection can be accomplished with sharp or cutting instruments (knives, scissors) or electrosurgical instruments. Microinstruments are available for microsurgery of the larynx and trachea, but these require handling by an experienced surgeon. Also, the same time, the division of tissues with cutting instruments always involves capillaries and small arterial or venous vessels, leading to diffuse bleeding at the surgical site. Although the volume of blood loss in the larynx is usually not an issue, the bleeding nevertheless obscures the operative field and makes it difficult to assess the progress of the resection, often slowing the procedure down. This type of bleeding is particularly troublesome in the situation of narrow margin microsurgery such as is the case in LD. No reliable data on the results of cold instrument surgery for LD have been published in recent years. Sadri et al. noted a pooled local control rate of 77% in their literature review [19]. A reasonable alternative to TLM may be the use of microdissection electrodes [40]. However, again there is a lack of large series comparable to those reported for TLM.

## Open surgery

The transoral approach is the main way to treat surgically LD. In particular cases; however, open surgery still can have a role. The situation and the discussion is pretty similar to that in T1a vocal fold carcinoma and the main indication is when there is not an adequate exposure for transoral surgery, for example in LD located at the level of the posterior commissure. Open surgery has the advantage of excellent access to the lesions and the resection can be adapted according to the dysplasia size.

Currently, the only indication for open partial resection in LD is the inadequate exposure by a conventional transoral approach. The surgical approach is usually performed via a midline thyrotomy. If needed, to have a better vision of the posterior larynx, the orotracheal intubation can be temporarily placed though the inferior part of the thyrotomy.

To our knowledge, there are not specific publications on results with open surgery in the particular setting of LD in the last 25 years due do the development of transoral surgery but the results can be expected similar to those obtained by transoral approach.

## Medical treatment and prevention

Smoking cessation is clearly the most important issue in the prevention of LD and of recurrences following initial treatment [41]. There is a great, unmet clinical need for treatments of LD that provide nonsurgical options to patients and treat the entire epithelial field. A treatment agent that convincingly and substantially reduces LD burden might also decrease invasive cancer risk and the subsequent need for surgical interventions.

For the time being, chemotherapy and immunotherapy are not an issue in LD. For the vast majority of all patients, there are currently no meaningful medical approaches to the disease. In patients with LD related to laryngeal papillomatosis, HPV vaccination, treatment with Cidofovir [42] and bevacizumab [43] may be considered on an individual basis.

Recently, both aspirin [44–46] and metformin [47–49] have emerged as promising medications for the prevention of dysplasia formation in man. A small case series has reported on promising observations in nondiabetic patients treated with metformin for recurrent LD lesions [49]. Clinical long-term results, however, are not available to date. Folate deficiency may be considered to be a factor predisposing to precancerous lesions, and dietary folate supplementation may prevent and reduce the emergence of cancer [50]

## Follow-up

It is accepted that the follow-up for patients treated for LD is an essential part of their care. The reasons of post-treatment follow-up in the setting of laryngeal cancer in general have been previously reported by Simo et al. [51]. However, controversy still exists in how these aims should be achieved, while increasing efforts are being made to rationalize the structure and timing of follow-up clinics to minimize pressure on the health care systems ensuring appropriate standard of care.

The length of follow-up has been described to be very variable. Cosway and Weller recently reported an evidence-based chart to aid clinicians with the follow-up of LD after excision [35, 52]. They recommended a minimum follow-up of 6 months for patients with low-grade or mild-to-moderate dysplasia with no risk factors, while a follow-up for 5 years in those with high-risk or severe dysplasia [35]. Follow-up for more than 5 years can be justified in those who have progressed or recurred after appropriate treatment, and for those who continue to smoke and drink heavily. Fear of recurrence is prevalent in cancer patients and continued attendance at clinic helps to mitigate this issue [53].

At present, there is no evidence that a high frequency of follow-up visits confers any benefit in terms of reducing morbidity and mortality. As a consequence, the majority of clinicians support a follow-up schedule with decreasing frequency overtime. In the first 2 years, when the risk of loco-regional recurrence is high, the frequency should remain high and be followed by a gradual decrease in the second year. Therefore, the follow-up in the first 2 years should be in between 4 and 8 weeks and from 3 to 6 months thereafter, especially when considering patients managed for a high-grade LD [35]

At present, most patients tend to be followed-up by their treating clinicians. While in most European countries follow-up is usually performed by Otolaryngologists, in the United Kingdom, introduction of clinical nurse specialists and the key worker role in management of patients with HNC, has become vital to open lines of communication between the patient and family, their careers, their general practitioner and the clinical team should any problems arise [51]. This could also be also extended to the management of LD.

Traditionally, clinical assessment has been the most important aspect of the follow-up in patients treated for LD. The clinical evaluation should include inspection of the larynx, employing the use of transoral rigid enedoscope or transnasal video- or fibroscopy and palpation of the neck. By focusing on vocal fold vibration during phonation, using VLS, the laryngologist can contribute considerably to the diagnosis of persistent/recurrent LD while differentiating this from attended scar tissues or other iatrogenic sequelae. The diagnostic accuracy of laryngeal imaging in general is up to 68%. Particular diagnoses, however, are more consistently identified; cancer, for example, was much more accurately identified on laryngoscopy (100%) and VLS (100%) than on history and physical examination alone (33%) [54]. Moreover, photo- and video documentation allows to compare changes, playing an important role also from an academic and patient’s educational point of view. There is little evidence that CT, MR, and PET scanning may play a role in the follow-up of LD unless there is a suspect of progression to invasive carcinoma.

Patient’s education is a key factor in the management of LD and its incorporation into follow-up symptom evaluation is to be recommended. As a matter of fact, the understanding of the potential symptoms and signs of recurrence is a key factor in early diagnosis and patients should be aware of these so they can seek medical attention as soon as they perceive any subtle modification of their voice, lasting more than 2–3 weeks. Patient’s education should also include tobacco smoking and alcohol cessation programmes, which have proven to be of great value for the prevention of progression and recurrence of LD. However, future behavioural change interventions should be conducted within populations with LD [55].

Evidence to support follow-up for early detection of LD progression or recurrence is lacking, although there is a common belief that follow-up clinics have inherent value in detecting morphological and progression changes. However, with the current socio-economic climate and with health care systems reducing costs, there is a real risk that long-term follow-up for patients with LD or even laryngeal cancer may be seriously affected in the near future. Therefore, it may be needed that health care professionals provide risk-assessment strategies to rationalize potential unnecessary follow-up, for example subdividing patients with LD into low- and high-risk of developing progression to invasive carcinoma. It is believed that this categorization could help to determine how and for how long patients should be followed for. It would also help to establish which screening tests might be needed to detect recurrence or progression to invasive cancer [22].

## New technologies in the follow-up

The ideal clinical investigation tool for patients for follow-up after treatment for LD is flexible nasopharyngolaryngoscope. Videorecording using high resolution flexible videoendoscopes is paramount for accurate documentation, archiving of images, minimising inter-observer discrepancies and allowing comparisons during the follow-up period. Narrow Band Imaging (NBI), with associated high definition television technology, can be an excellent adjunctive imaging tool. This is owed to its specific capability to selectively address superficial persistent changes/recurrences or second primary tumors by enhancing their pathognomonic neoangiogenetic pattern. It has been reported that its use can detect 18% more true positive laryngeal cancer lesions than conventional white light (WL) endoscopy. This has been also demonstrated after RT, due to the high accuracy (98%) of NBI in differentiating between neoplastic disease and post-RT inflammatory and/or cicatricial changes [56, 57].

A recent systematic review by Paleri et al. demonstrated that with a histological diagnosis of mild dysplasia which show no abnormalities on NBI, the post-test probability of malignancy is 2.3% compared to 10.3 with conventional WL imaging. For severe dysplasia, similar post-test probabilities after NBI and WL are 8.0 and 29.7%, respectively. Post-test probabilities in this setting indicate the chance of missing malignancy following NBI or WL in patients who undergo no further intervention [34].

Recently a specific Image Enhancement System (SPIES) [5] has been accessible to clinicians; however, no robust data have been reported yet. However, the possible advantages of its use in detection of recurrent/persistent or progressing LD may be similar to those already described for NBI, due to the overlapping information obtainable with such a device.

## Conclusion

The diagnosis of LD largely relies on endoscopic procedures and on histopathology. Diagnostic efficiency of endoscopy may be improved using VLS and bioendoscopic tools such as NBI and SPIES. Current histological classifications are not powerful enough to clearly predict the risk to carcinoma evolution and technical issues such as sampling error, variation in epithelial thickness and inflammation hamper pathological examination. Almost all dysplasia grading systems differ but are effective in different ways. The 2017 WHO system should prove to be an improvement as it is slightly more reproducible and easier for the non-specialist pathologist to apply. To optimize treatment decisions, surgeons should know how their pathologist grades samples and preferably audit their transformation rates locally. Whether carcinoma in situ should be used as a classification remains contentious and pathologists should agree with their clinicians whether they find this additional grade useful in treatment decisions. Recently, different studies have defined the possible utility of different biomarkers in risk classification. The main treatment modality for LD is TLM. RT may be indicated in specific circumstances such as multiple recurrence or wide-field lesions. Medical treatment currently does not have a significant role in the management of LD. Follow-up for patients treated with LD is a fundamental part of their care and investigations may be supported by the same techniques used during diagnosis (VLS and NBI/SPIES).
